# Supplementary material for: Active Inference and Epistemic Value in Graphical Models
Source: Front Robot AI. 2022 Apr 6;9:794464. doi: 10.3389/frobt.2022.794464 (PMC9019474; doi:10.3389/frobt.2022.794464)
Supplement: Supplementary file 1 [file DataSheet1.pdf]

Active Inference and Epistemic Value in Graphical Models: Supplementary Material

Thijs van de Laar [1,*], **Magnus Koudahl** [1,2], **Bart van Erp** [1] **and Bert de Vries** [1,3]

[1] *Department of Electrical Engineering, Eindhoven University of Technology, Eindhoven, The Netherlands*
[2] *Nested Minds Solutions, Liverpool, England*
[3] *GN Hearing Benelux BV, Eindhoven, The Netherlands*

Correspondence*:
Thijs van de Laar
t.w.v.d.laar@tue.nl

APPENDIX A. EVALUATION OF THE EXPECTED FREE ENERGY

The standard procedure for evaluating the Expected Free Energy (EFE) collects instantaneous EFE contributions over time by a forward filtering approach (Friston et al., 2015). Following (Friston et al., 2015; Da Costa et al., 2020), the EFE constructs an instantaneous model for each future time-point $\tau \geq t$, as

$$f(y_\tau, x_\tau | \boldsymbol{u}_{t:\tau}) = p(x_\tau | y_\tau, \boldsymbol{u}_{t:\tau})\, \tilde{p}(y_\tau)\,, \tag{1}$$

with $\tilde{p}(y_\tau)$ the goal prior, and $p(x_\tau|y_\tau, \boldsymbol{u}_{t:\tau})$ a state posterior that needs to be further defined.

Using Bayes rule, we can express the state posterior in terms of the observation model and a posterior predictive for the state, as

$$\begin{aligned} p(x_\tau|y_\tau, \boldsymbol{u}_{t:\tau}) &= \frac{p(x_\tau|\boldsymbol{u}_{t:\tau}) p(y_\tau|x_\tau)}{p(y_\tau|\boldsymbol{u}_{t:\tau})} && \text{(2a)}\\ &= \frac{p(x_\tau|\boldsymbol{u}_{t:\tau}) p(y_\tau|x_\tau)}{\sum_{x_\tau} p(x_\tau|\boldsymbol{u}_{t:\tau}) p(y_\tau|x_\tau)}\,. && \text{(2b)} \end{aligned}$$

The posterior predictive $p(x_\tau|\boldsymbol{u}_{t:\tau})$ is explicitly conditioned on the policy $\boldsymbol{u}_{t:\tau}$, from current time t up to and including future time τ, and thus represents the forward prediction (filtering solution) for the current state belief given preceding controls (whilst excluding preceding goals). Using the generative model engine definition, the posterior predictive for the state then becomes[1]

$$\begin{aligned} p(x_\tau|\boldsymbol{u}_{t:\tau}) &= \sum_{\boldsymbol{y}_{t:\tau}} \sum_{\boldsymbol{x}_{t-1:\tau-1}} p(x_{t-1}) \prod_{k=t}^{\tau} p(y_k, x_k | x_{k-1}, u_k) && \text{(3a)}\\ &= \sum_{\boldsymbol{x}_{t-1:\tau-1}} p(x_{t-1}) \prod_{k=t}^{\tau} p(x_k|x_{k-1}, u_k)\,. && \text{(3b)} \end{aligned}$$

[1] The definition of (Friston et al., 2015, p. 192) implicitly defines this forward prediction as a marginalization over states, which is made explicit in the definition of (3). In general, this marginalization need not be tractable, in which case it can also be approximated by on-line optimization of an appropriate BFE objective on the generative model engine.

The second step of (3) simplifies the expression by marginalizing over $\boldsymbol{y}_{t:\tau}$. The posterior predictive can then conveniently be computed by message passing on the generative model (Loeliger, 2002), using a single forward pass.

We are now prepared to construct the instantaneous EFE (the EFE at time τ), which is defined as (Friston et al., 2015)

$$\mathrm{G}_\tau(\hat{\boldsymbol{u}}_{t:\tau}) = \mathbb{E}_{p(y_\tau|x_\tau)\,p(x_\tau|\hat{\boldsymbol{u}}_{t:\tau})}\left[\log \frac{p(x_\tau|\hat{\boldsymbol{u}}_{t:\tau})}{f(y_\tau, x_\tau|\hat{\boldsymbol{u}}_{t:\tau})}\right] . \tag{4}$$

Upon substitution of (2a) in (4), the instantaneous EFE factorizes into ambiguity and risk, as

$$\begin{aligned}
\mathrm{G}_\tau(\hat{\boldsymbol{u}}_{t:\tau}) &= \mathbb{E}_{p(y_\tau|x_\tau)\,p(x_\tau|\hat{\boldsymbol{u}}_{t:\tau})}\left[\log \frac{p(y_\tau|\hat{\boldsymbol{u}}_{t:\tau})}{p(y_\tau|x_\tau)\,\tilde{p}(y_\tau)}\right] \\
&= -\mathbb{E}_{p(x_\tau|\hat{\boldsymbol{u}}_{t:\tau})}\left[\mathbb{E}_{p(y_\tau|x_\tau)}[\log p(y_\tau|x_\tau)]\right] + \mathbb{E}_{p(y_\tau|x_\tau)\,p(x_\tau|\hat{\boldsymbol{u}}_{t:\tau})}\left[\log \frac{p(y_\tau|\hat{\boldsymbol{u}}_{t:\tau})}{\tilde{p}(y_\tau)}\right] \\
&= \underbrace{\mathbb{E}_{p(x_\tau|\hat{\boldsymbol{u}}_{t:\tau})}[\mathrm{H}[p(y_\tau|x_\tau)]]}_{\text{ambiguity}} + \underbrace{\mathrm{KL}[p(y_\tau|\hat{\boldsymbol{u}}_{t:\tau})\|\tilde{p}(y_\tau)]}_{\text{observation risk}} .
\end{aligned} \tag{5}$$

This decomposition is often used to compute the instantaneous EFE in practice.

The complete EFE of the full policy $\hat{\boldsymbol{u}}$ then follows by summation of all instantaneous contributions

$$\mathrm{G}(\hat{\boldsymbol{u}}) = \sum_{\tau=t}^{t+T-1} \mathrm{G}_\tau(\hat{\boldsymbol{u}}_{t:\tau}) . \tag{6}$$

To summarize, the procedure for computation of the EFE in practice (Friston et al., 2015; Da Costa et al., 2020) usually consists of three steps. First, for a given policy $\hat{\boldsymbol{u}}$, the posterior predictive distributions (3) are computed for all $t \leq \tau < t + T$. Then, the instantaneous EFE's are (individually) computed. Finally, the instantaneous EFE's are summed to produce the full-policy EFE (6).

REFERENCES

Da Costa, L., Parr, T., Sajid, N., Veselic, S., Neacsu, V., and Friston, K. (2020). Active inference on discrete state-spaces: a synthesis. *arXiv:2001.07203 [q-bio]* ArXiv: 2001.07203

Friston, K., Rigoli, F., Ognibene, D., Mathys, C., Fitzgerald, T., and Pezzulo, G. (2015). Active inference and epistemic value. *Cognitive Neuroscience* 6, 187–214. doi:10.1080/17588928.2015.1020053

Loeliger, H.-A. (2002). Least Squares and Kalman Filtering on Forney Graphs. In *Codes, Graphs, and Systems*, eds. R. E. Blahut and R. Koetter (Boston, MA: Springer US), vol. 670. 113–135. doi:10.1007/978-1-4615-0895-3_7
